# Xenograft of Human Umbilical Mesenchymal Stem Cells Promotes Recovery from Chronic Ischemic Stroke in Rats

**DOI:** 10.3390/ijms23063149

**Published:** 2022-03-15

**Authors:** Yu-Show Fu, Chang-Ching Yeh, Pei-Ming Chu, Wen-Hsing Chang, Maan-Yuh Anya Lin, Yung-Yang Lin

**Affiliations:** 1 Department of Anatomy and Cell Biology, School of Medicine, National Yang Ming Chiao Tung University, Taipei 112304, Taiwan; ysfu@nycu.edu.tw; 2 Department of Obstetrics and Gynecology, Taipei Veterans General Hospital, Taipei 112201, Taiwan; ccyeh39@gmail.com; 3 Department of Obstetrics and Gynecology, National Yang Ming Chiao Tung University, Taipei 112304, Taiwan; 4 Department of Nurse-Midwifery and Women Health, National Taipei University of Nursing and Health Sciences, Taipei 112303, Taiwan; 5 Department of Anatomy, School of Medicine, China Medical University, Taichung 404328, Taiwan; pmchu@mail.cmu.edu.tw; 6 Institute of Anatomy and Cell Biology, School of Medicine, National Yang Ming Chiao Tung University, Taipei 112304, Taiwan; katteken@gmail.com; 7 Department of Medical Research, Taipei Veterans General Hospital, Taipei 112201, Taiwan; 8 Department of Pharmacy, National Yang Ming Chiao Tung University, Taipei 112304, Taiwan; 9 Neurological Institute, Taipei Veterans General Hospital, Taipei 112201, Taiwan; 10 Department of Critical Care Medicine, Taipei Veterans General Hospital, Taipei 112201, Taiwan; 11 Institute of Brain Science, National Yang Ming Chiao Tung University, Taipei 112304, Taiwan; 12 Institute of Clinical Medicine, National Yang Ming Chiao Tung University, Taipei 112304, Taiwan

**Keywords:** chronic stroke, cerebral ischemia, umbilical mesenchymal stem cells, Wharton’s jelly, xenograft, transplantation, neuroprotection, angiogenesis, neurologic deficit, MRI

## Abstract

Stroke is a leading cause of adult disability. In our previous study, transplantation of human umbilical mesenchymal stem cells (HUMSCs) in Wharton’s jelly in the acute phase of ischemic stroke promotes recovery in rats. Unfortunately, there is no cure for chronic stroke. Patients with chronic stroke can only be treated with rehabilitation or supportive interventions. This study aimed to investigate the potential of xenograft of HUMSCs for treating chronic stroke in rats. Rats were subjected to 90 min middle cerebral artery occlusion and then reperfusion to mimic ischemic cerebral stroke. On day 14 following stroke, HUMSCs were transplanted into the damaged cerebral cortex. The motor function in rats of the Stroke + HUMSCs group exhibited significant improvement compared to that of the Stroke + Saline group, and the trend persisted until day 56 post stroke. The cerebral cortex changes were tracked using magnetic resonance imaging, showing that cerebral atrophy was found starting on day 7 and was reduced significantly in rats receiving HUMSCs compared to that in the Stroke + Saline group from day 21 to day 56. HUMSCs were found to be existed in the rats’ cerebral cortex on day 56, with signs of migration. The grafted HUMSCs did not differentiate into neurons or astrocytes and may release cytokines to improve neuroprotection, decrease inflammation and increase angiogenesis. Our results demonstrate that xeno-transplantation of HUMSCs has therapeutic benefits for chronic ischemic stroke. Most importantly, patients do not need to use their own HUMSCs, which is a gospel thing for clinical patients.

## 1. Introduction

Stroke is among the leading causes of death and disability in adults [1]. In 2019, over 10 million new cases are reported globally, and over 6 million died due to cerebral stroke [2]. Ischemic stroke incidents comprise nearly 90% of all strokes [1]. The mean survival post stroke is 6 to 7 years, and indeed more than 85% of patients live past the first year post stroke, many with years of enduring disability and neuropsychiatric syndrome [3,4,5,6]. Once entering the chronic stage of stroke, the patient’s welfare is affected, and significant family and societal burdens are experienced. To date, there are no effective drugs or therapies available for the treatment of chronic stroke; treatment mainly relies on rehabilitation or supportive treatments to prevent extensive tissue loss and further degeneration. Therefore, it is particularly important to identify a new approach in treating chronic ischemic stroke.

Mesenchymal stem cells (MSCs) are among the leading restorative therapy candidates [7,8]. As a part of medical waste after delivery, the umbilical cord can be more easily retrieved than embryos or bone marrow. Based on our previous studies, transplantations of umbilical mesenchymal stem cells in the Wharton’s jelly (HUMSCs) into the striatum, spinal cord, cerebral cortex, hippocampus, and cerebellum can effectively treat Parkinson’s disease, [9,10] spinal cord injury [11], acute stroke [12], epilepsy [13], and cerebellum atrophy [14]. In addition to central nervous system diseases, we have investigated the feasibility of the HUMSCs transplantation for the treatment of liver fibrosis, peritoneum fibrosis, pulmonary fibrosis, osteoporosis, and diabetes [15,16,17,18,19,20,21]. The results demonstrated that xenograft of the HUMSCs did not cause immune reaction or rejection in the host. Therefore, we believe that HUMSCs can be used as transplantation materials for clinical medicine.

In our previously published results, we performed ligation of the middle cerebral artery for 90 min and reperfusion in rats in order to trace the changes in the cerebral cortex with continuous MRI imaging. Severe edema was observed in the cerebral cortex on day 1 to day 2 post stroke. The cerebral cortex exhibits severe atrophy on day 14 post stroke. Therefore, we suspected that it had entered chronic stroke on day 14 after surgery-induced cerebral ischemia and reperfusion. Furthermore, at 24 h post stroke, i.e., the acute stage of stroke, transplantation of HUMSCs into the rat’s infarcted region of the cerebral cortex significantly alleviated the level of atrophy in the injured brain region and improved its mobility. The results suggested that HUMSCs transplantation can be applied for the treatment of acute stroke [12]. Currently, there is no cure for chronic stroke. However, on day 14 post stroke, i.e., the chronic stage of stroke, signs of atrophy were already displayed in the cerebral cortex. Whether HUMSCs transplantation performed on day 14 post stroke could still exhibit the treatment effect needs to be further elucidated in this study.

## 2. Results

### 2.1. Ischemia–Reperfusion Surgery Causes Cortical Infarction

**Figure 1:**
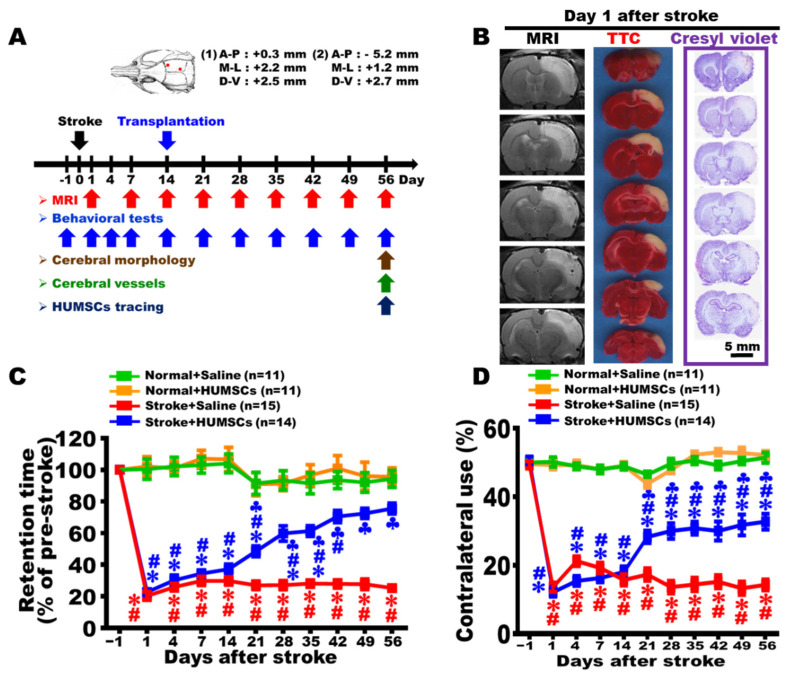
HUMSCs transplantation enhanced motor function in rats with chronic stroke. (**A**) The diagram shows the time course for various experiments in this study. Representative MRI, TTC-stained, and cresyl violet stained images of rat brains are shown on day 1 after stroke, showing that right cerebral cortices were markedly enlarged (**B**). The Stroke + HUMSCs group show better functional recovery in the rotarod test (**C**) and cylinder test (**D**) than the Stroke + Saline group. Normal + Saline *n* = 11, Normal + HUMSCs *n* = 11, Stroke + Saline *n* = 15, Stroke + HUMSCs *n* = 14. ∗, vs. the Normal + Saline group at the same day, *p* < 0.05. #, vs. the Normal + HUMSCs group at the same day, *p* < 0.05. ♣, vs. the Stroke + Saline group at the same day, *p* < 0.05.

As shown by MRI images, TTC staining, and Cresyl violet staining, the infarcted cortex of the rats in the Stroke + Saline group were considerably swollen and edematous 1 day post stroke. These images indicated that an ischemia–reperfusion stroke model was successfully established in our experiments (Figure 1B).

### 2.2. HUMSCs Transplantation Improves Motor Function in Rats with Chronic Stroke

The results of the rotarod test showed that administration of saline, or HUMSCs in the rats’ right cortices of the Normal + Saline and Normal + HUMSCs groups caused only a minor change in retention time, without statistical significance. The retention time was significantly reduced to 21–23% of the normal level in the Stroke + Saline and Stroke + HUMSCs groups 1 day post stroke, indicating the mobility was impaired by cerebral ischemia. The situation persisted until day 14 post stroke. On day 21 post stroke, the Stroke + Saline group had a retention time of 27% of the normal level, and the declining trend remained until day 56 post stroke without showing any improvement. On day 7 after stem cell transplantation, i.e., day 21 post stroke, the retention time of the Stroke + HUMSCs group increased to 49%, a significant elevation compared to that in the Stroke + Saline group. The statistical trend sustained until day 56 post stroke. Although the retention time in the Stroke + HUMSCs group increased (from day 21 to day 42), it was not as long as the time obtained in those of the groups of Normal + Saline and Normal + HUMSCs (*p* < 0.05) (Figure 1C and Supplemental Table S1).

The quantitative results of the cylinder test showed that the contralateral use of forelimb of the four groups were approximately 50% prior to the surgery, i.e., under normal situation. In the Normal + Saline and Normal + HUMSCs groups, even with injections of saline or HUMSCs into the right cerebral cortex on day 14, contralateral use of forelimb did not statistically change and maintain until day 56 (Figure 1D and Supplemental Table S2).

On day 1 post stroke, the left forelimb activity was substantially reduced compared to pre-stroke, with a percentage of 12–14%, indicating that stroke caused impaired left forelimb activity. The situation remained until day 14 post stroke. On day 21 post stroke, left forelimb use in the Stroke + Saline group was around 17%, which was significantly lower than those of Normal + Saline and Normal + HUMSCs groups. The activity was not improved but sustained until day 56 post stroke (*p* < 0.05) (Figure 1D and Supplemental Table S2). In the Stroke + HUMSCs group, the contralateral use elevated to 28.29 ± 1.84% on day 7 after transplantation (i.e., day 21 post stroke), which was a significant increase compared to that in the Stroke + Saline group, but was statistically reduced compared to those in Normal + Saline and Normal + HUMSCs groups. The trend remained until day 56 post stroke (*p* < 0.05) (Figure 1D, Supplemental Table S2 and Supplemental Videos S1–S3).

### 2.3. HUMSCs Transplantation Reduces Brain Atrophy in Rats with Chronic Stroke as Assessed by MRI

**Figure 2:**
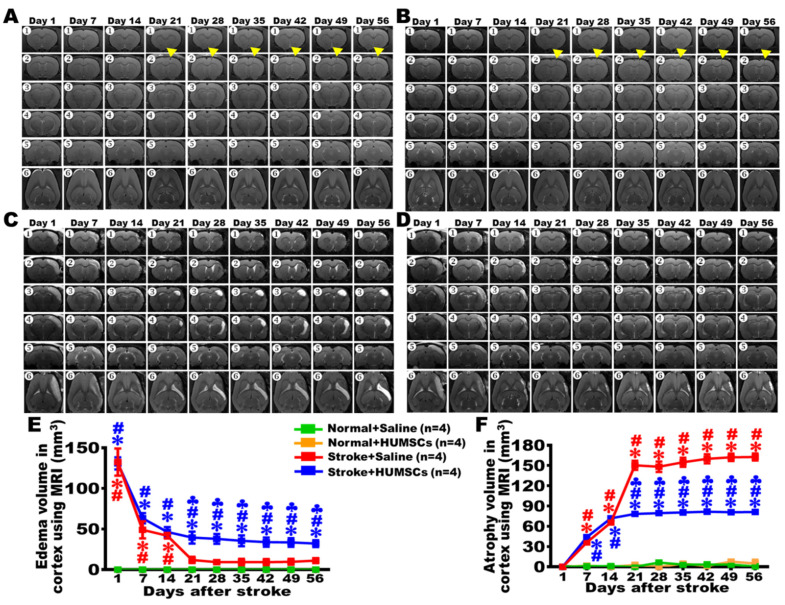
Cortical edema and atrophy examined by MRI. Brain MRI examination was conducted on day 1 (➊–➏), day 7 (➊–➏), day 14 (➊–➏), day 21 (➊–➏), day 28 (➊–➏), day 35 (➊–➏), day 42 (➊–➏), day 49 (➊–➏), and day 56 (➊–➏). Along the rostral–caudal orientation, coronal slices (➊–➎) and horizontal slices (➏) were taken for monitoring the alterations of brain damages in these rats. MRI slices of the Normal + Saline (**A**) and Normal + HUMSCs (**B**) groups did not find significant edema or atrophy areas from day 1 to day 56. MRI slices of the Stroke + Saline group (**C**) and the Stroke + HUMSCs group (**D**) were obtained on day 1 post stroke. White areas in the right cerebral cortex were seen, indicating severe inflammation and edema occurred in this region (➊–➏). On day 7 and day 14, there was a reduction in the white edema cortices, but slight atrophy had appeared in the right infarcted area when comparing to the left cerebral cortex (➊–➏). The volumes of edema (**E**) and atrophy (**F**) in the right cerebral cortex were quantified post stroke. Atrophy of the infarcted cortex becomes apparent in the Stroke + Saline group but is significantly less severe in the Stroke + HUMSCs group from day 21 to day 56. The results of brain MRI revealed that HUMSCs transplantation alleviated cerebral atrophy in rats with chronic stroke. Normal + Saline *n* = 4, Normal + HUMSCs *n* = 4, Stroke + Saline *n* = 4, Stroke + HUMSCs *n* = 4. ∗, vs. the Normal + Saline group at the same day, *p* < 0.05. #, vs. the Normal + HUMSCs group at the same day, *p* < 0.05. ♣, vs. the Stroke + Saline group at the same day, *p* < 0.05. 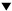 indicates the first injection site.

The alterations in the infarcted cerebral cortex at different time points were observed using MRI. The results showed that injections of saline, or HUMSCs in the rats’ right cortices of the Normal + Saline and Normal + HUMSCs groups did not caused any significant edema or atrophy. The trend remained until day 56 (Figure 2A,B,E,F, and Supplemental Tables S3 and S4).

The Stroke + Saline group displayed a significant white area, suggesting that severe inflammation and edema were present in the cerebral cortex on day 1. The volume of the cortical edema decreased on day 7 and day 14. Since day 21, edema of the injured cerebral cortex markedly reduced, the situation remained until day 56 post stroke. The injured cerebral cortex of the Stroke + Saline group began showing signs of atrophy on day 7 post stroke. From day 21 onwards, the volume of atrophy further increased; the trend sustained until day 56 (Figure 2C,E,F and Supplemental Tables S3 and S4). We postulate that edema areas switched into atrophy areas without treatment; edema areas of the injured cerebral cortex markedly reduced at day 21, at the same time, atrophy area in the Stroke + Saline group was increased. It is a worthy of attention that the right lateral ventricle significantly enlarged and showed signs of edema from day 21 to day 56 in the Stroke + Saline group.

The Stroke + HUMSCs group had similar patterns of cerebral cortex edema with the Stroke + Saline group from day 1 to day 14 post stroke. On day 7 after transplantation (day 21 post stroke), the volume of edema and atrophy in the cerebral cortex showed no significant changes compared with those in day 14. However, there was a statistical difference when comparing to that in the Stroke + Saline group at the same time point; the trend remained until day 56. The edema of the right lateral ventricular was also milder than that in the Stroke + Saline group (Figure 2D–F and Supplemental Tables S3 and S4). These results demonstrate that HUMSCs transplantation has a protective role in preventing the infarct cortex from atrophy post stroke.

### 2.4. HUMSCs Transplantation Preserves Cerebral Cortex in Rats with Chronic Stroke

**Figure 3:**
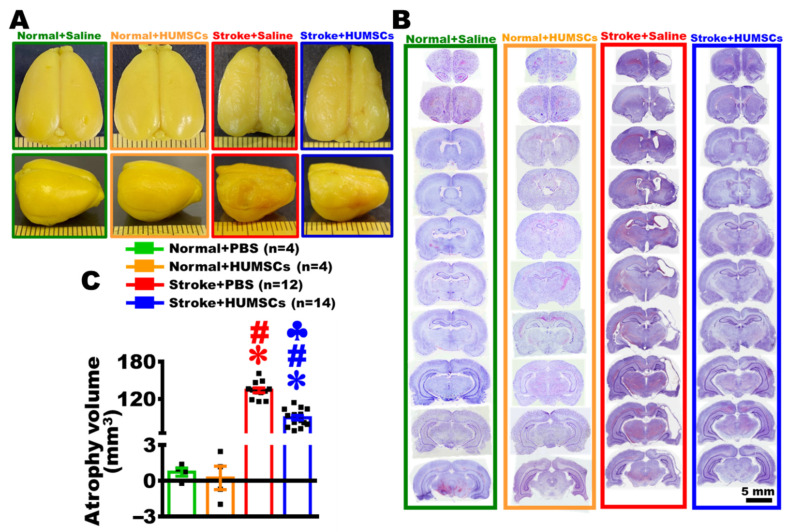
HUMSCs transplantation preserved the cerebral cortex in rats with chronic stroke. On day 56 post stroke, the brains fixed with paraformaldehyde were photographed from a superior (top panel) and a lateral view (bottom panel). The brain of the Stroke + Saline group exhibited more severe atrophy in the cortex than that grafted with HUMSCs (**A**). Subsequently, the brains of four groups were subjected to serial coronal sections (from bregma +2.52 mm to −5.76 mm) and stained with cresyl violet to observe alterations in the cerebral cortex. The results showed that the brain grafted with HUMSCs had more preserved cortices (**B**). The volume of atrophy in the infarcted cortex was quantified using the sections stained by cresyl violet, the result indicated the atrophy volume of the Stroke + HUMSCs group was substantially lower than that of the Stroke + Saline group (**C**). Normal + Saline *n* = 4, Normal + HUMSCs *n* = 4, Stroke + Saline *n* = 12, Stroke + HUMSCs *n* = 14. ∗, vs. the Normal + Saline group, *p* < 0.05. #, vs. the Normal + HUMSCs group, *p* < 0.05. ♣, vs. the Stroke + Saline group, *p* < 0.05. ■ is the value of individual experiment.

We likewise used gross appearance and brain sections with cresyl violet staining to determine the therapeutic effect of HUMSCs on the infarct cortex of chronic stroke. On day 56 post stroke, the brains were intact and smooth in the Normal + Saline and Normal + HUMSCs groups. The gross appearance of the Stroke + Saline group had a marked indentation at the right cerebral cortex with extensive cortex atrophy. Although the HUMSCs group had an apparent shrinkage at the right infarcted cerebral cortex, it was less severe than that in the Stroke + Saline group (Figure 3A).

On day 56, cresyl violet staining showed that both of the right and left cerebral cortices were intact in the Normal + Saline and Normal + HUMSCs groups. From the coronal brain sections of the Stroke + Saline group, not only the cerebral cortex had severe atrophy, but the corpus callosum, basal ganglia, and hippocampus also showed signs of atrophy. In addition, brain sections showed an enlarged right lateral ventricle on day 56 post stroke. Although the coronal cerebral sections of the Stroke + HUMSCs group also showed signs of atrophy, these signs were primarily restricted to the regions of the cerebral cortex (Figure 3B). By summing all quantitative data from serial brain sections stained by cresyl violet, it showed that the volume of right cerebral cortex was similar to that of left cerebral cortex in the Normal + Saline and Normal + HUMSCs groups. The overall volumes of right cerebral atrophy were 133.88 ± 3.90 mm^3^ and 89.69 ± 3.72 mm^3^ in the Stroke + Saline and Stroke + HUMSCs groups, respectively. Moreover, the volume of cerebral atrophy in the Stroke + HUMSCs group was also remarkably lower than that in the Stroke + Saline group (*p* < 0.05) (Figure 3C and Supplemental Table S5).

### 2.5. HUMSCs Transplantation Helps Neuronal Cell Survival in the Infarcted Brain in Rats with Chronic Stroke

**Figure 4:**
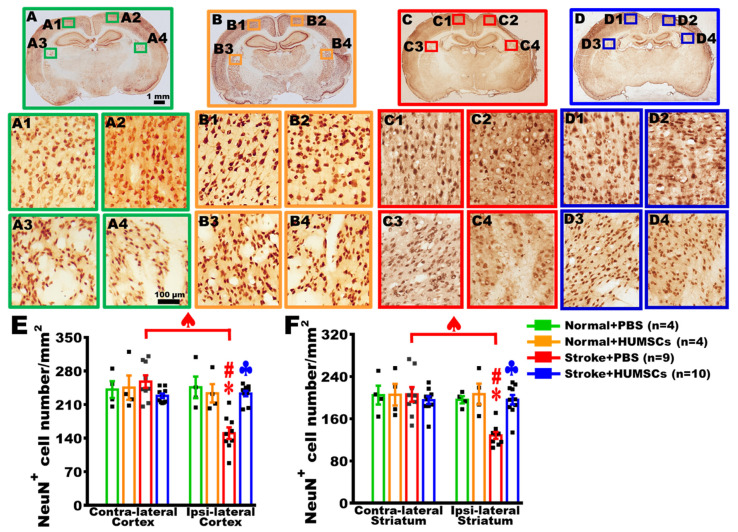
HUMSCs transplantation increased the survival of neuronal cells in the infarcted brain in rats with chronic stroke. On day 56 post stroke, anti-NeuN immunohistochemical staining was applied to observe the number of survived neuronal cells. Brain coronal sections at bregma −1.92 mm are displayed ((**A**): Normal + Saline group; (**B**): Normal + HUMSCs group; (**C**): Stroke + Saline group; (**D**): Stroke + HUMSCs group). For high magnification, the peripheral region to the infarcted cerebral cortex (**A2**,**B2**,**C2**,**D2**) or striatum (**A4**,**B4**,**C4**,**D4**) are shown, as well as undamaged contralateral sides of the cerebral cortex (**A1**,**B1**,**C1**,**D1**) and striatum (**A3**,**B3**,**C3**,**D3**). The number of NeuN^+^ cells in the cerebral cortex (**E**) and striatum (**F**) was quantified. The results showed that the neuronal cell numbers in the cerebral cortex and striatum peripheral to the damaged brain areas were significantly higher in the Stroke + HUMSCs group than those in the Stroke + Saline group. Normal + Saline *n* = 4, Normal + HUMSCs *n* = 4, Stroke + Saline *n* = 9, Stroke + HUMSCs *n* = 10. ∗, vs. the ipsilateral region of Normal + Saline group, *p* < 0.05. #, vs. ipsilateral region of the Normal + HUMSCs group, *p* < 0.05. ♣, vs. ipsilateral region of the Stroke + Saline group, *p* < 0.05. ♠, contra-lateral vs. ipsi-lateral region in the Stroke + Saline group, *p* < 0.05. ■ is the value of individual experiment.

On day 56 post stroke, coronal brain sections were subjected to anti-NeuN immunohistochemical staining to observe the alterations of neuronal cells localized at the peripheral regions of the infarcted areas in the cerebral cortex and striatum. The results found that the neuronal cells in the cerebral cortex and striatum exhibited relatively healthy morphology in the Normal + Saline and Normal + HUMSCs groups (Figure 4A(A1–A4) and Figure 4B(B1–B4)). Even with injections of saline or HUMSCs into the right cerebral cortex, the neuronal number in the right cerebral cortex or striatum was not statistically different from that of the contralateral regions in the Normal + Saline and Normal + HUMSCs groups (Figure 4E,F and Supplemental Table S6).

The results showed that the neuronal cells in the cerebral cortex and striatum surrounding the infarcted region exhibited a lighter and relatively loose staining in the Stroke + Saline group (Figure 4C,(C1–C4)). The neuronal number in the infarction cerebral cortex (ipsilateral cortex) of the Stroke + Saline group was significantly lower than those in the Normal + Saline and Normal + HUMSCs groups. Additionally, the number of neuronal cells in the cerebral cortex surrounding the infarcted region was significantly lower than the normal contralateral location (*p* < 0.05). Similarly, the number of neuronal cells in the damaged striatum also statistically decreased when comparing to the normal contralateral side of the brain (*p* < 0.05) (Figure 4E,F and Supplemental Table S6).

In the cerebral cortex and striatum surrounding the infarcted area of the Stroke + HUMSCs group, the numbers of neuronal cells were preserved at a higher level when comparing to those in the ipsilateral regions of Stroke + Saline group (*p* < 0.05). Furthermore, no statistical differences were found in the number of neuronal cells when comparing to the contralateral cerebral cortex and striatum (*p* > 0.05) (Figure 4D(D1–D4),E,F and Supplemental Table S6).

### 2.6. HUMSCs Transplantation Promotes Angiogenesis in the Infarcted Brain in Rats with Chronic Stroke

**Figure 5:**
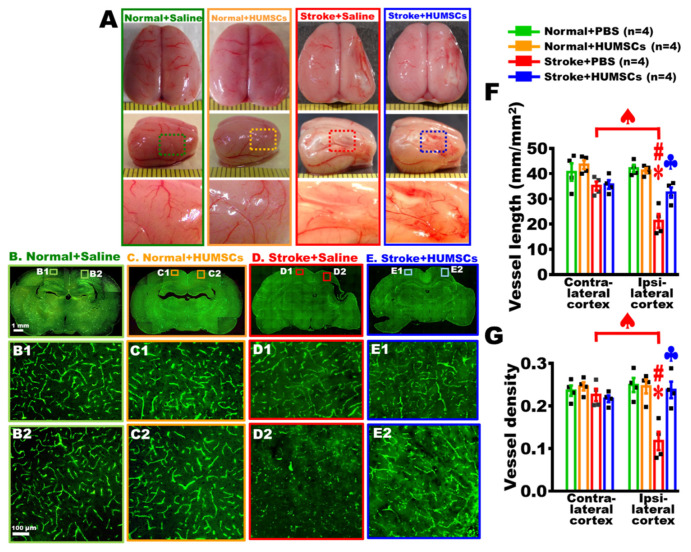
HUMSCs transplantation promoted angiogenesis in the infarcted brain of the rats with chronic stroke. On day 56 post stroke, the brains were photographed from a superior view (top panel) and a lateral view (intermediate panel and high magnification in the bottom panel) to observe the distribution of superficial blood vessels of the cerebral cortex. More blood vessels are observed on the surface of the Stroke + HUMSCs group (**A**). From coronal brain sections at bregma −2.4 mm, vessels in the contralateral cortex (**B1**,**C1**,**D1**,**E1**) and ipsilateral cortex (**B2**,**C2**,**D2**,**E2**) are visualized by fluorescein isothiocyanate-conjugated dextran amine. Boxed areas in the photographs are magnified in the bottom row (**B1**,**B2**,**C1**,**C2**,**D1**,**D2**,**E1**,**E2**). The total vessel length per mm^2^ (**F**) and vessel density (**G**) in the infarcted region are significantly greater in the cortex of the Stroke + HUMSCs group than those in the Stroke + Saline group. Normal + Saline *n* = 4, Normal + HUMSCs *n* = 4, Stroke + Saline *n* = 4, Stroke + HUMSCs *n* = 4. ∗, vs. ipsilateral cortex of the Normal + Saline group, *p* < 0.05. #, vs. ipsilateral cortex of the Normal + HUMSCs group, *p* < 0.05. ♣, vs. ipsilateral cortex of the Stroke + Saline group, *p* < 0.05. ♠, contra-lateral vs. ipsi-lateral cortex in the Stroke + Saline group, *p* < 0.05. ■ is the value of individual experiment.

On day 56 post stroke, the brains of each group were freshly obtained to observe the distribution of superficial blood vessels of the brain. The qualitative observations showed that the blood vessels with small size were apparent in the Normal + Saline and Normal + HUMSCs groups. In the Stroke + Saline group, the right hemisphere markedly shrunk, with fewer blood vessels found on the surface of the injured cerebral cortex. The vessels with small diameter were observed in the Stroke + HUMSCs group (Figure 5A).

Additionally, brain sections with perfused by FITC-dextran to quantify vascular distribution near the infarction area. The results showed that blood vessels had a regular reticular pattern in both the right and left cerebral cortices of the Normal + Saline and Normal + HUMSCs groups, as well as in the normal left cerebral cortices of the Stroke + Saline and Stroke +HUMSCs groups (Figure 5B(B1,B2),C(C1,C2),D(D1),E(E1)). The quantitative results indicated that the total vessel lengths were around 43.56 ± 1.73 to 35.09 ± 1.78 mm/per mm^2^, and the vessel density was between 0.25 ± 0.01 to 0.22 ± 0.0 in the normal cerebral cortices of Normal + Saline, Normal + HUMSCs, Stroke + Saline and Stroke + HUMSCs groups (Figure 5F,G and Supplemental Table S7).

In the brain area surrounding the right damaged cerebral cortex of the Stroke + Saline group, the reticular distribution of the brain vessels displayed a destructed pattern, with the total vessel length significantly shortened to 21.25 ± 2.79 mm/per mm^2^ and the vessel density markedly reduced to 0.12 ± 0.02, which were significantly lower than the normal left contralateral side (Figure 5(D2),F,G and Supplemental Table S7). In the Stroke + HUMSCs group, the number of vessels in the brain area surrounding the right damaged cerebral cortex was higher than that in the Stroke + Saline group, although not as regular or reticular as under normal conditions. There were also many small, thin blood vessels shown (Figure 5(E2),F,G and Supplemental Table S7). The quantitative results showed that the total vessel length and density in the peripheral region surrounding the right damaged cerebral cortex in the Stroke + HUMSCs group were both statistically increased when comparing to those in the corresponding region of the Stroke + Saline group (*p* < 0.05). Furthermore, the total vessel length and density in the ipsilateral cortex of the Stroke + HUMSCs group were similar to those in the Normal + Saline and Normal + HUMSCs groups (Figure 5F,G and Supplemental Table S7). It indicated that HUMSCs may promote angiogenesis in the ischemic cortex of the chronic stroke.

### 2.7. Engrafted HUMSCs Survive and Migrate in the Infarcted Cortex of Rats with Chronic Stroke

**Figure 6:**
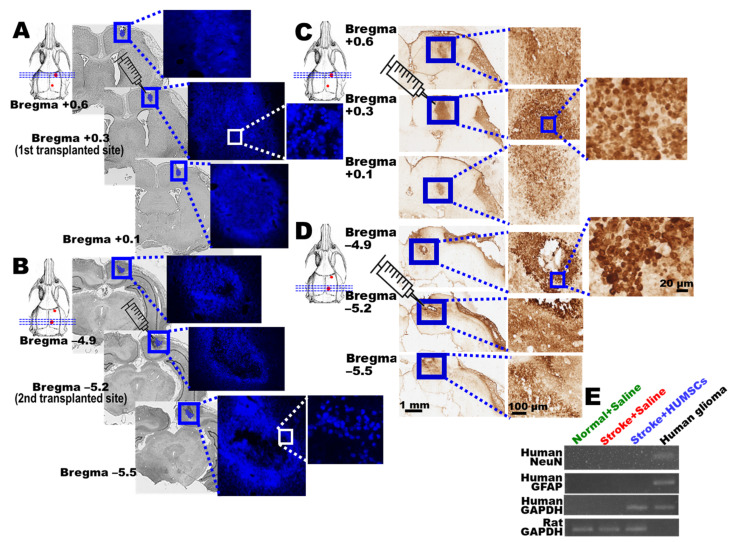
Grafted HUMSCs survived in the infarcted cortex and did not differentiate into neuronal cells or astrocytes**.** The nucleus of HUMSCs was labeled with bisBenzimide (blue), and the cells were transplanted into the rat’s right cerebral cortex on day 14 post stroke. On day 56, phase-contrast microscopy images of the HUMSCs group are displayed with the section at the first HUMSCs transplantation position of bregma +0.3 mm and its anterior (+0.6 mm) and posterior coronal sections (+0.1 mm). The blue clusters of cells were observed at high magnification (**A**). The second HUMSCs transplantation position at bregma −5.2 mm and its anterior (bregma −4.9 mm) and posterior (bregma −5.5 mm) coronal brain sections are shown. At high magnification, clusters of cells labeled with bisBenzimide were found (**B**). Immunostaining for human-specific nucleus antigen demonstrates the aggregation of HUMSCs around the first (**C**) and secondary (**D**) transplantation sites at 56 days post stroke. The boxed areas in the left column are magnified in the right column. On day 56 post stroke, the cerebral cortex was subjected to RT-PCR tests to examine whether human NeuN or GFAP could be detected in all the groups. The results showed that the transplanted HUMSCs did not differentiate into neuronal cells or astrocytes (**E**).

The nucleus of HUMSCs was labeled with bisBenzimide to track their survival and distribution. On day 56, the fluorescence imaging showed that plenty of blue clusters of cells were found at bregma +0.3 mm, i.e., the first site for HUMSCs transplantation, in the Stroke + HUMSCs group. From bregma +0.6 mm to +0.1 mm, there were still many cells in blue fluorescence identified (Figure 6A). At the second transplantation site (bregma −5.2 mm) of the Stroke + HUMSCs group, large clusters of cells could also be found. Similarly, HUMSCs existed in clusters from bregma −4.9 mm to bregma −5.5 mm (Figure 6B).

Anti-human-specific nuclei antigen immunohistochemical staining was further employed to investigate HUMSCs distribution in rats with chronic stroke. The results showed that large clusters of HUMSCs were seen at the first and secondary stem cell transplantation sites of the cerebral cortex in the Stroke + HUMSCs group (at bregma +0.3 mm and −5.2 mm). The number of HUMSCs gradually decreased as the distance away from the implanted sites increased rostrally or caudally (Figure 6C,D). No signal was detected in the cerebral cortex of the Stroke + Saline group (data not shown).

### 2.8. HUMSCs Does Not Differentiate into Neurons and Astrocytes in Rats with Chronic Stroke

On day 56 post stroke, rat brains were freshly obtained and examined using human NeuN and GFAP primers to elucidate whether the HUMSCs grafted had differentiated into neuronal cells or astrocytes in the cerebral cortex of rats with chronic stroke. The results showed that genes of human NeuN or GFAP were not detected in the cerebral cortex of the Stroke + HUMSCs group, indicating HUMSCs may not have differentiated into neuronal cells or astrocytes. Human glioma cells served as a positive control for human NeuN or human GFAP (Figure 6E).

## 3. Discussion

In the present study, on day 14 post stroke, when significant atrophy was identified in the injured brain region of the SD rats (i.e., the chronic stage of stroke), the infarcted brain tissues were obviously preserved, and the mobility was markedly improved following transplantations of HUMSCs.

The questions about the intervention timing, cell dosage, and route of administration for stem cell therapy on stroke have been in heated discussions [22,23,24,25,26]. HUMSCs (3 × 10^5^–1 × 10^7^) were intravenously injected into rats on days 1, 7, 30, and 90 post stroke, and the brain tissue sections were examined on day 140. The results showed that there was no statistical difference between all the treatment groups and the control group in terms of the cerebral injury volume. However, for the group transplanted with ≥3 × 10^6^ HUMSCs, though the macroscopic morphology of the cerebral cortex did not exhibit a significant change, the mobility was significantly improved compared to that in the control group, suggesting that the amount of HUMSCs transplanted played a crucial role [27]. Another study also evaluated the beneficial treatment effects of 3 × 10^7^ bone marrow mononuclear cells (BMMCs) that were injected via the jugular vein of the rats on days 1, 7, 14, or 30 post stroke. The results showed that the groups transplanted with BMMCs on days 14 or 30 exhibited similar behaviors to that of the control group, suggesting that transplantation performed during the acute phase was superior to the chronic phase [22]. Ishizaka et al. transplanted bone marrow-derived mesenchymal stem cells (BMMSCs) into rats via the internal carotid artery on 1, 4, or 7 days post stroke. The results showed that for the group receiving transplantation on day 7, only a few BMMSCs were found distributed over the brain, and therefore no apparent sensorimotor function recovery was observed in the rats. The results indicated that the reservation of the cerebral cortex was much better compared to that in the control group only in the group receiving transplantation on day 1 [24]. Although the origins of stem cells varied, their results indicated that stem cell transplantation in the acute phase of stroke can reduce neurological deficits and improve functional recovery [12,28,29,30,31,32,33,34,35]. In fact, although some cerebral ischemia patients really survived, they struggled with the impasse of chronic stroke. Currently, there is no cure for chronic stroke. Therefore, it is a therapeutic imperative to find the treatment for chronic stroke.

Given the aforementioned study results, we suspected the number of the survived stem cells in the infarcted brain region plays a critical role in recovery. At the chronic stage of stroke, we suggest that the types or the concentration of the chemotactic factors attracting the stem cells circulating in the blood to the infarcted brain region have relatively decreased or recessed compared to those at the acute stroke stage. Therefore, to treat chronic stroke, direct intra-cerebral transplantation of the stem cells into the brain infarction region might be a more effective treatment approach. The present study’s results also demonstrated that a large number of the transplanted HUMSCs survived in the rats’ cerebral cortex until day 56 post stroke. However, intra-cerebral implantation is an invasive method which is never recommended to be used in clinical medicine. Therefore, the route of stem cell transplantation may be modified, such as applying stem cells on the surface of the cerebral cortex.

Although the stem cells’ types might be different, the treatment mechanisms can be generally categorized into two directions. First, the transplanted stem cells differentiate into neuronal cells to replace the injured or dead neurons [36,37,38]. Secondly, the transplanted stem cells alter the injured environment via the cytokines secreted. The secreted cytokines might possess the effects of neuroprotection [12,39,40,41], neuroregeneration [42,43], angiogenesis [12,41,42,43,44], or immunomodulation [39,45,46,47], and thereby repair the damaged tissues or prevent them from further deterioration. From the RT-PCR results of our study, it is suggested that HUMSCs did not directly differentiate into neurons or astrocytes after transplantation into the infarcted cortex of the chronic stroke rats. We suggested that implanted HUMSCs may secrete cytokines to improve neuronal survival, increase angiogenesis and enhance functional recovery. Interestingly, HUMSCs may release different cytokines in response to the pathophysiological microenvironments accordingly [11,12,13,14,15,17]. As demonstrated in our previous study, implanted HUMSCs did not differentiate into neurons but released neuroprotective and growth-associated cytokines, including neutrophil-activating protein-2 (NAP-2), angiopoientin-2, brain-derived neurotrophic factor, CXCL-16, and platelet-derived growth factor-AA in the infarcted cortex of acute ischemia stroke [12]. Instead of differentiating into neurons, astrocytes, or oligodendrocytes, transplanted HUMSCs released neurotrophin-3, NAP-2, basic fibroblast growth factor, glucocorticoid-induced tumor necrosis factor receptor family, and vascular endothelial growth factor receptor-3 in the transected spinal cord [11]. ImplantedHUMSCs survived in the epileptic hippocampus and released FGF-6, amphiregulin, glucocorticoid-induced tumor necrosis factors receptor (GITR), MIP-3β, and osteoprotegerin [13]. At the same time, human IL-13, GIF, PAI-1, FGF-2, and CXCL-4 were significantly increased in the cerebella of SCA1 mice [14]. Furthermore, without differentiating into alveolar epithelial cells or hepatocytes, HUMSCs grafted into rat fibrotic lung and liver release a considerable amount of human FGF-6 and IGF-1 in lung and human prolactin, leukemia inhibitory factor, and cutaneous T cell-attracting chemokine in livers [15,17].

The therapeutic effect of stem cell species on stroke have also been investigated. Yu et al. administered both auto-adipose derived mesenchymal stem cell (auto-ADMSCs) and allo-ADMSCs in an experimental animal model of stroke to compare their effectiveness. Their results demonstrated that auto-ADMSCs were more effective than allo-ADMSCs to promote recovery and reduce the infarct volume of stroke rats. The improved effects of auto-ADMSCs are likely related to decreased immunological responses, which thereby result in higher survival rates, longer survival times, a wider migratory space, and a lower amount of apoptosis [48]. However, divergent opinions have been raised. Gutiérrez-Fernández et al. suggested that human AD-MSCs and rat AD-MSCs showed equal efficacy in terms of functional recovery and decreased ischemic brain damage in rat [49]. Similarly, autologous and allogeneic cell therapy for ischemic heart disease show a similar improvement in left ventricular ejection fraction in large animal models of myocardial ischemia [50]. HUMSCs were used in this study. The HUMSCs are collected from the umbilical cord, which is generally considered to be a waste after childbirth and can be obtained without invasive techniques. The amount of HUMSCs collected can be amplified greatly in cultures. Moreover, xeno-transplanted HUMSCs are immunologically compatible in recipients [9,10,11,12,13,14,15,16,17,18,19,20]. Therefore, HUMSCs can serve as an excellent stem cell source for treatment of the chronic stroke in clinical medicine. 

In this study, our results showed that on day 56 post stroke, HUMSCs transplanted into the cerebral cortex did not differentiate into neuronal cells or astrocytes. It also showed that an increased number of neuronal cells was preserved, angiogenesis was facilitated, and animal behavior was promoted; therefore, we suggest that the HUMSCs ameliorated and treated chronic stroke by secreting cytokines. 

## 4. Materials and Methods

### 4.1. Experimental Animals

Sprague Dawley (SD) rats used in the present study were provided by the Laboratory Animal Center of National Yang-Ming University. Rats were housed in transparent polycarbonate (PC) cages (size: 45 × 24 × 20 cm) under a 12/12 h reversed light/dark cycle (lights on from 8:00 a.m. to 8:00 p.m.) and constant temperature (22 ± 2 °C), with bedding changed weekly. The rats were allowed ad libitum access to food and water.

### 4.2. Procedures of Middle Cerebral Artery Occlusion (MCAO) and Reperfusion

Male Sprague Dawley rats (weighing 280 to 320 g) were used in this study. The rats were anesthetized with Zoletil 20–40 mg/kg and Xylazine 5–10 mg/kg (intraperitoneal injection) and fixed on a stereotaxic frame. We performed MCAO surgery and reperfusion to induce cerebral infarction by ligating the right middle cerebral artery and bilateral common carotid arteries, as previously described [12]. Briefly, a curved, 1 cm skin incision was made vertically by the right orbit, and the temporalis muscle was removed to uncover the junction of the zygomatic arch and the squamous bone, where a 3 mm^2^ burr hole was drilled to expose the middle cerebral artery. The artery was ligated with a 10–0 suture while both common carotid arteries were clamped simultaneously by nontraumatic aneurysm clips for 90 min, after which the ligature and clips were removed to restore blood flow. Subsequently, the incision sites were sutured and disinfected, and the rats were placed on a heating mat until recovery from anesthesia.

### 4.3. Isolation and Culture of Human Umbilical Mesenchymal Stem Cells (HUMSCs)

The mesenchymal tissue in Wharton’s jelly was then diced into cubes 0.5 cm on each side and centrifuged at 250 g for 5 min. After removal of the supernatant fraction, the precipitate that contained mesenchymal tissue was washed with serum-free Dulbecco’s Modified Eagle’s Medium (DMEM) and centrifuged at 250 g for 5 min. After aspiration of the supernatant fraction, mesenchymal tissue in the precipitate was treated with 0.0125% type I collagenase solution (Sigma-Aldrich, St. Louis, MO, USA) at 37 °C for 18 h, washed, and further digested with 0.025% trypsin at 37 °C for 30 min. The treated umbilical tissue then was cultured until the HUMSCs migration and proliferation. Finally, HUMSCs were stored in liquid nitrogen for later transplantation. HUMSCs were collected between the 10th and 15th passages for transplantation into rats in this study.

### 4.4. Transplantation of HUMSCs

HUMSCs were transplanted into the right cerebral cortex in rats suffering a stroke. Two transplantation sites were targeted with a stereotaxic instrument: first coordinates for transplantation: AP = −0.3 mm, R/L = +2.2 mm, H = +2.7 mm; second coordinates for transplantation: AP = −5.8 mm, R/L = +1.2 mm, H = + 2.5 mm. On day 14 post stroke, atrophy of the right cerebral cortex was observed. The coordinates were chosen because the two transplantation sites were located at the outer regions of the shrunken cortex. An amount of 5 × 10^5^ HUMSCs were drawn into a glass pipette with a tip diameter of 150–200 µm mounted onto a Hamilton syringe (Hamilton, Reno, NV) attached to a micromanipulator. A total of 5 × 10^5^ HUMSCs were transplanted at each site, and a total of 10^6^ cells were administered for each rat. For the stroke group, 5 μL PBS was injected at each location (Figure 1A).

### 4.5. BisBenzimide-Treated HUMSCs In Vitro

Cultured HUMSCs were treated with 1 μg/mL bisBenzimide (Sigma B2883) for 48 h to label the cell nucleus. Subsequently, HUMSCs were treated with trypsin, collected, and transplanted into the right cerebral cortex at two locations on day 14 post stroke. On day 56 following a stroke, rats were sacrificed and perfused. The brains were obtained and immersed in 30% sucrose buffer until they sunk to the bottom. Protected from light, the samples were subjected to serial cryosectioning. Finally, the sections were observed and photographed using fluorescence microscopy.

### 4.6. Experimental Groups

There were four groups as follows:

1. Normal + Saline group (*n* = 12): The rats did not receive MCAO and reperfusion, just remove their skulls. On day 14, no treatment was administered except the injection of normal saline into the rat’s cerebral cortex.
2. Normal + HUMSCs group (*n* = 12): The rats did not receive MCAO and reperfusion, just remove their skulls. On day 14, HUMSCs were transplanted into the rat’s cerebral cortex.
3. Stroke + Saline group (*n* = 26): The rats received MCAO and reperfusion. On day 14 post-MCAO, no treatment was administered except the injection of normal saline into the rat’s cerebral cortex.
4. Stroke + HUMSCs group (*n* = 26): The rats received MCAO and reperfusion. HUMSCs were transplanted into the rat’s cerebral cortex on day 14 after MCAO.

The four groups were subjected to various assessments at different time points following MCAO (Figure 1A).

### 4.7. Infarct Cortex Identification

Rats were deeply anesthetized and decapitated. Coronal sections of the brains were sliced at 2 mm, immersed in 2% 2,3,5-triphenyltetrazolium chloride (TTC) (T8877; Sigma), and then fixed with 10% formalin. The infarct area was devoid of red staining.

### 4.8. Magnetic Resonance Imaging (MRI)

Brain images were acquired using Bruker S300 BioSpec/MedSpec MRI instrument at the Instrumentation Center, National Taiwan University. Normal brain was normally grey; brain images with white signals suggest high water/fluid contains, i.e., brain edema.

The four groups (Normal + Saline, Normal + HUMSCs, Stroke + Saline, and Stroke + HUMSCs groups) were assessed with MRI at nine time points, on days 1, 7, 14, 21, 28, 35, 42, 49, and 56, to continuously monitor the changes in brain damages caused by chronic stroke. Five brain images around bregma +0.48 mm, −0.72 mm, −1.92 mm, −3.12 mm, and −4.32 mm was chosen and summed for quantification. The MRI images of each group were quantified using image processing software Image-Pro Plus to quantify the area of cerebral cortex edema (white areas) in each image. The edema volume of the cerebral cortex was obtained by summing up white area in right cerebral cortex and multiplying the thickness of the section (0.6 mm). Subsequently, the atrophy area of the right cerebral cortex was obtained by subtracting the area of the injured right cerebral cortex from the area of the left (unaffected) cerebral cortex. The atrophy volume of the cerebral cortex was obtained by summing up all areas and multiplying the thickness of the section (0.6 mm).

### 4.9. Behavioral Test

Two behavioral tests were involved in the present study, including the cylinder test and the rotarod test. 

### 4.10. Cylinder Test

Rats were put in a transparent cylinder (20 cm in diameter × 30 cm in height). A camera was placed toward the bottom of the cylinder for video recording to observe the percentages of each forelimb touching the cylinder wall while standing with hindlimbs. Normal rats generally use each forelimb with a frequency of approximately 50%, suggesting that usage of right and left forelimb are almost symmetrical. In this study, rats suffering a stroke in the right cerebral hemisphere caused locomotor deficits of their left bodies. Hence, the times of touching cylinder wall with left forelimb were divided by the total number of touches using two forelimbs to obtain the frequency of left forelimb usage. The use of impaired contralateral forelimb should significantly decrease in rats with stroke, according to previous studies [12].

### 4.11. Rotarod Test

Rats were placed on the rotarod with an initial speed of 4 rpm. The speed of the rotarod gradually accelerated to 40 rpm within 5 min. The time that each rat stayed on the rod will be recorded to assess its motor coordination and balance [12]. The retention time of each rat before receiving MCAO was set as a baseline value of 100%. By dividing the retention time obtained post stroke with the retention time obtained before the stroke, the ratio of the retention time in percentage was obtained. Stroke causes impairments of rats’ mobility, which is reflected by the reduced duration they stay on the rotarod.

The two behavioral tests of the four groups were performed at 11 time points: 1 day prior to stroke (−1) and days 1, 4, 7, 14, 21, 28, 35, 42, 49, and 56 post stroke.

### 4.12. Numbering Brain Cryosections

Rats were anesthetized and perfused transcardially with 4% paraformaldehyde. Frozen sections were serially sectioned at 30 μm in a cryostat and stained with cresyl violet and immunocytochemistry staining for light microscopy (Supplemental Figure S1).

### 4.13. Cresyl Violet Staining

Brain sections were stained with 1% cresyl violet solution for 5 min, followed by passing through a series of increasing alcohol concentrations (50%, 70%, 80%, 90%, 95%, and 100%) for dehydration. Slides were then submerged twice in 100% xylene for 5 min each. Finally, slides were mounted with a mounting medium (Fisher Scientific SP15-500) and photographed under an optical microscope. The area of the normal left cerebral cortex of each section was quantified using the software Image pro-plus. By subtracting the area of the damaged cerebral cortex of the right hemisphere from the area of the normal left cerebral cortex and multiplying by 30 μm (thickness of the tissue section), the atrophy volume of the cerebral cortex was obtained. Subsequently, the total atrophy volume of the entire brain was gained by summing up the volume of all sections stained with cresyl violet.

### 4.14. Immunohistochemical (IHC) Staining

Brain sections were reacted with primary antibodies of mouse anti-neural nuclei antigen (NeuN) antibody (Millipore, 1:250), mouse anti-ED1 antibody (Millipore, 1:500), or mouse anti-human-specific nuclei antigen antibody (Millipore, 1:100) at 4 °C for 12 h. Subsequently, the secondary antibody of goat anti-mouse IgG-conjugated biotin (Millipore, 1:250) was added and reacted for 1 h at room temperature. Slides were then washed with 0.1 M PBS three times (5 min each), reacted with avidin-biotinylated complex (ABC kit, Vector) for 1 h, washed three times with 0.1 M PBS (5 min each), and developed with DAB. After staining, the slides were mounted with a mounting medium (Fisher Scientific SP15-500) and examined and photographed under an optical microscope.

### 4.15. Counting the Number of Neuronal Cells

Immunohistochemically stained with anti-NeuN antibody, the number of neuronal cells per mm^2^ within the cerebral cortex and striatum in right infarcted brain regions of five brain slides at bregma +0.48 mm, −0.72 mm, −1.92 mm, −3.12 mm, and −4.32 mm was quantified. Four fields (×20 objective lens) were selected at each brain section. The four areas along the infarcted cortex were chosen for cell counting for the cerebral cortex and the four areas below the corpus callosum were chosen for cell counting for the striatum. Additionally, the numbers of neurons within the normal contralateral regions of the cerebral cortex and striatum were also quantified to serve as a reference.

### 4.16. Perfusion of the Experimental Animals with Fluorescein Isothiocyanate-Dextran (FITC-dextran)

Following anesthesia, the rats were injected with 0.2 mL fluorescein isothiocyanate-dextran (FITC-dextran; 50 mg/mL, Sigma FD-2000S) through the left ventricle [12]. Two minutes later, rat brain was obtained and placed in 4% paraformaldehyde fixation solution for 24 h. For dehydration, the tissues were then submerged in 30% sucrose until the tissue sunk to the bottom. Subsequently, the brain was covered with cryo-embedding media and fixed on the cryostat for sectioning under −20 °C. The brain tissues were sectioned into 30 µm-thick slices, enabling observation, and brain vessel distribution was photographed using fluorescence microscopy.

Five brain sections at bregma +0.48 mm, −0.72 mm, −1.92 mm, −3.12 mm, and −4.32 mm, labeled with FITC-dextran, were analyzed using Image pro-plus. Along the edges of the infarcted region in the right cerebral cortex, four fields (×20 objective lens) were selected at each brain section. The four fields next to the infarcted cortex were chosen for green fluorescence counting to measure the total length of FITC-dextran^+^ blood vessels, representing the total length of the blood vessels per mm^2^. In addition, the percentage of FITC-dextran^+^ area was quantified to represent the density of blood vessels. The contralateral cerebral cortex without injury served as a corresponding reference.

### 4.17. Reverse Transcription-Polymerase Chain Reaction (RT-PCR)

Total RNA of the cerebral cortex was extracted using TRIzol^®^ reagent (Invitrogen^®^ 15596018). After quantification using a spectrophotometer, a total of 5 μg RNA was subjected to reverse transcription (Invitrogen^®^). Subsequently, 2 μL cDNA was used for PCR with the following primers:
**HUMAN RBFOX3:**310 bpF: 5′-ATCCAGTGGTCGGCGCAGTCTAC-3′
R: 5′-TACGGGTCGGCAGCTGCGTA-3′
**Human GFAP:**122 bpF:5′-CTGGAGAGGAAGATTGAGTCGC-3′
R: 5′-ACGTCAAGCTCCACATGGACCT-3′
**Rat GAPDH:**160 bpF: 5′-CTCTACCCACGGCAAGTTCAAC-3′
R: 5′-GGTGAAGACGCCAGTAGACTCCA-3′
**Human GAPDH:**176 bpF: 5′-TCCTCCACCTTTGACGCT-3′
R: 5′-CTTCCTCTTGTGCTCTTG-3′


The temperature setting was as follows: denature for 1 min at 95 ℃; annealing for 1 min at 59 ℃; extension for 1 min at 72 ℃. After 35 cycles, the PCR products were analyzed by agarose gel electrophoresis and visualized and photographed with a UV transilluminator.

### 4.18. Statistical Analysis

All data are presented as the mean ± SEM. Comparisons of the means were performed using one-way ANOVA. Multiple comparisons were further conducted with Tukey’s test. *p <* 0.05 was considered statistically significant.

## 5. Conclusions

HUMSCs transplanted into the cerebral cortex of rats with chronic stroke did not differentiate into neurons or astrocytes. Recovery or preservation of the infarcted region in rats with chronic stroke might be mediated via the cytokines secreted by the transplanted HUMSCs, which protected the neuronal cells and promoted angiogenesis in the infarcted region. These results are important for the design of future clinical trials.

## Supplementary Materials

The following supporting information can be downloaded at: https://www.mdpi.com/article/10.3390/ijms23063149/s1.
