# Supplementary material for: Xenograft of Human Umbilical Mesenchymal Stem Cells Promotes Recovery from Chronic Ischemic Stroke in Rats
Source: Int J Mol Sci. 2022 Mar 15;23(6):3149. doi: 10.3390/ijms23063149 (PMC8953545; doi:10.3390/ijms23063149)
Supplement: Supplementary file 1 [file ijms-23-03149-s001.zip › ijms-1629434-supplementary/Figure S1.pdf]

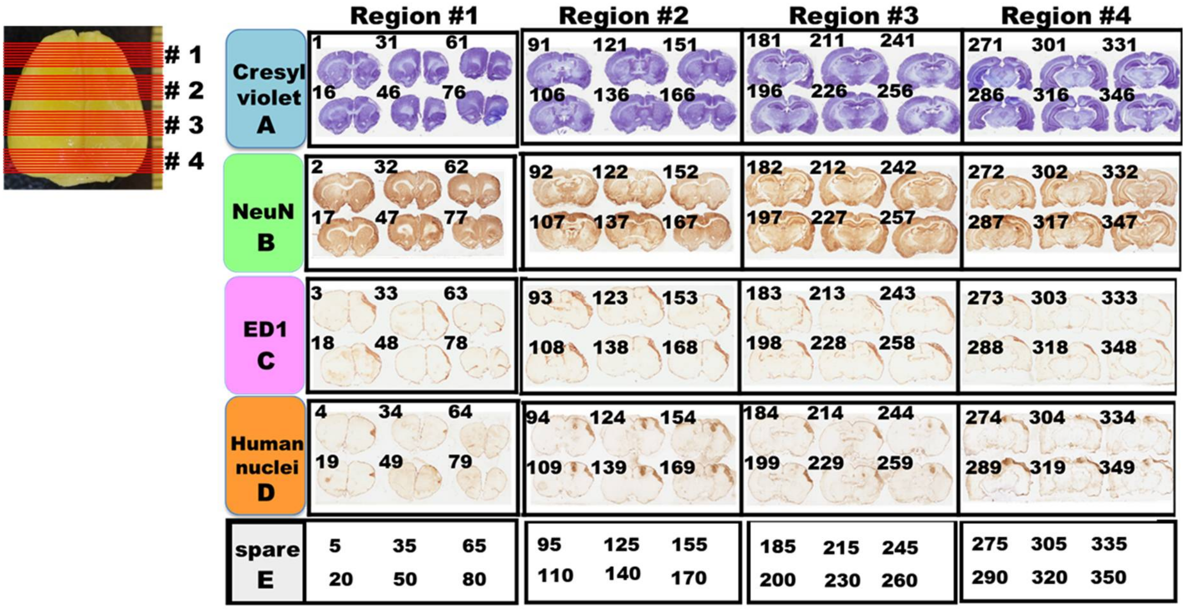

Supplemental Figure S1. Brain tissue sectioning methodology for morphology/pathology analysis in chronic stroke rat. Rat brains from each group were subjected to serial coronal sections in 30 μm-thick using a cryostat. Five consecutive coronal sections numbered 1, 2, 3, 4, and 5 were obtained started from bregma +4.2 mm and onward, which were placed on slides lettered A, B, C, D, and E. Subsequently, the next 10 consecutive slices were discarded. The following 10 consecutive slices numbered 16, 17, 18, 19, and 20 were then placed on slides lettered A, B, C, D, and E. For region #1, the same procedures were repeated until section numbered 80 was obtained and placed on a slide. Sections were subjected to various immunohistochemical stainings for observing the pathological conditions, the number of neuronal cells, and distribution of HUMSCs within region #1. Similarly, the procedures of sectioning and staining were repeated until section numbered 350 was collected at around bregma −5.8 mm. From anterior to posterior, the brain sections could be divided into four regions (#1 to #4), which enabled observation of alterations in the entire cerebral cortex. The first row’s slices represent the brain sections from regions #1 to #4 placed on the first slide, which was subjected to cresyl violet staining to observe any pathological changes. The second row’s slices represent the brain sections from regions #1 to #4 placed on the second slide, which was subjected to immunohistochemical staining using anti-neural nuclei antigen (NeuN) to observe changes of neuronal cells. The third row’s slices represent the brain sections from regions #1 to #4 placed on the third slide, which was subjected to immunohistochemical staining using anti-ED1 to observe changes of microglia. The fourth row’s slices represent the brain sections from regions #1 to #4 placed on the fourth slide, which was subjected to immunohistochemical staining using anti-human-specific nuclei antigen to investigate the distribution of HUMSCs. The fifth row’s slices represent the brain sections from regions #1 to #4 placed on the fifth slide, which was preserved as spares.
