# Supplementary material for: Xenograft of Human Umbilical Mesenchymal Stem Cells Promotes Recovery from Chronic Ischemic Stroke in Rats
Source: Int J Mol Sci. 2022 Mar 15;23(6):3149. doi: 10.3390/ijms23063149 (PMC8953545; doi:10.3390/ijms23063149)
Supplement: Supplementary file 1 [file ijms-23-03149-s001.zip › ijms-1629434-supplementary/Tables S1-S7.pdf]

Supplement Table S1. Rotarod test

Days after stroke	Normal+Saline (n=11)	Normal+HUMSCs (n=11)	Stroke+Saline (n=15)	Stroke+HUMSCs (n=14)
-1	100.00±0.00	100.00±0.00	100.00±0.00	100.00±0.00
1	100.47±6.39	102.17±6.21	20.39±2.76 ✱#	22.75±2.08 ✱#
4	102.19±5.74	100.95±4.51	25.92±2.35 ✱#	30.28±3.32 ✱#
7	103.05±5.12	107.00±5.36	29.82±2.84 ✱#	34.48±2.23 ✱#
14	104.09±5.96	106.64±7.56	29.82±2.57 ✱#	37.28±3.20 ✱#
21	91.47±7.01	90.97±7.28	27.01±2.70 ✱#	48.57±3.38 ✱#♣
28	93.33±6.09	91.00±4.41	27.18±2.89 ✱#	59.81±4.81 ✱#♣
35	91.47±6.65	96.62±6.61	28.17±2.30 ✱#	61.32±3.42 ✱#♣
42	93.56±5.36	100.93±8.17	27.93±2.65 ✱#	70.51±3.82 #♣
49	92.06±6.16	96.05±8.79	27.71±3.32 ✱#	72.48±3.22 ♣
56	94.30±5.39	95.67±5.59	25.35±1.94 ✱#	75.58±3.42 ♣

✱ vs. the Normal+Saline group at the same day, $p < 0.05$

# vs. the Normal+HUMSCs group at the same day, $p < 0.05$

♣ vs. the Stroke+Saline group at the same day, $p < 0.05$

Supplement Table S2. Contralateral use (%)

Days after stroke	Normal+Saline (n=11)	Normal+HUMSCs (n=11)	Stroke+Saline (n=15)	Stroke+HUMSCs (n=14)
-1	50.00±1.24	49.56±1.42	49.33±1.03	50.57±1.21
1	50.18±1.41	49.27±1.30	13.60±1.37 ✱#	12.14±1.32 ✱#
4	48.91±0.87	49.27±1.33	21.20±1.42 ✱#	15.43±1.46 ✱#
7	48.00±1.18	48.00±1.18	19.20±1.57 ✱#	16.29±1.29 ✱#
14	48.91±1.02	49.09±1.12	15.73±1.65 ✱#	18.00±1.68 ✱#
21	46.45±0.68	43.79±1.18	17.30±1.81 ✱#	28.29±1.84 ✱#♣
28	49.68±1.47	47.82±0.47	13.60±1.85 ✱#	30.00±2.26 ✱#♣
35	50.56±1.10	52.27±0.70	14.40±1.96 ✱#	30.83±1.90 ✱#♣
42	49.16±1.18	53.04±0.59	15.20±1.95 ✱#	30.00±2.76 ✱#♣
49	50.44±0.94	52.79±1.64	13.27±1.93 ✱#	31.78±3.06 ✱#♣
56	51.40±1.54	52.16±1.27	14.27±1.58 ✱#	32.73±2.31 ✱#♣

✱ vs. the Normal+Saline group at the same day, $p < 0.05$

\# vs. the Normal+HUMSCs group at the same day, $p < 0.05$

♣ vs. the Stroke+Saline group at the same day, $p < 0.05$

Supplement Table S3. Edema volume in cortex using MRI

Days after stroke	Normal+Saline (n=4)	Normal+HUMSCs (n=4)	Stroke+Saline (n=4)	Stroke+HUMSCs (n=4)
1	0.00±0.00	0.00±0.00	132.40±16.73 ✱#	130.82±7.54 ✱#
7	0.00±0.00	0.00±0.00	49.12±10.50 ✱#	63.30±6.29 ✱#
14	0.00±0.00	0.00±0.00	41.83±3.33 ✱#	46.42±5.98 ✱#
21	0.00±0.00	0.00±0.00	11.96±3.82	39.55±7.31 ✱#♣
28	0.00±0.00	0.00±0.00	9.35±1.61	37.89±6.01 ✱#♣
35	0.00±0.00	0.00±0.00	9.45±3.87	35.52±6.64 ✱#♣
42	0.00±0.00	0.00±0.00	9.35±3.70	33.97±5.63 ✱#♣
49	0.00±0.00	0.00±0.00	9.72±3.62	33.51±5.34 ✱#♣
56	0.00±0.00	0.00±0.00	11.14±3.05	32.14±4.17 ✱#♣

✱ vs. the Normal+Saline group at the same day, $p < 0.05$

\# vs. the Normal+HUMSCs group at the same day, $p < 0.05$

♣ vs. the Stroke+Saline group at the same day, $p < 0.05$

Supplement Table S4. Atrophy volume in cortex using MRI

Days after stroke	Normal+Saline (n=4)	Normal+HUMSCs (n=4)	Stroke+Saline (n=4)	Stroke+HUMSCs (n=4)
1	0.00±0.00	0.00±0.00	0.00±0.00	0.00±0.00
7	0.92±1.46	1.66±0.68	36.24±1.52 ✱#	44.03±2.21 ✱#
14	1.18±1.66	-0.93±1.43	65.65±2.68 ✱#	71.90±1.67 ✱#
21	-2.49±4.20	2.84±3.09	150.33±6.76 ✱#	78.40±2.67 ✱#♣
28	6.42±1.52	-1.12±1.40	148.24±7.75 ✱#	79.62±2.53 ✱#♣
35	3.42±2.28	4.78±2.27	154.89±6.72 ✱#	80.58±3.07 ✱#♣
42	3.41±3.24	1.50±3.93	159.99±6.71 ✱#	81.72±3.06 ✱#♣
49	3.34±0.87	7.73±1.83	162.26±5.57 ✱#	80.86±2.92 ✱#♣
56	0.65±1.03	4.96±5.55	162.70±5.22 ✱#	81.22±2.81 ✱#♣

✱ vs. the Normal+Saline group at the same day, $p < 0.05$

# vs. the Normal+HUMSCs group at the same day, $p < 0.05$

♣ vs. the Stroke+Saline group at the same day, $p < 0.05$

Supplement Table S5. Atrophy volume by cresyl violet staining brain section

Group / mm^3	Normal+Saline (n=4)	Normal+HUMSCs (n=4)	Stroke+Saline (n=12)	Stroke+HUMSCs (n=14)
**Atrophy volume**	0.73±0.34	0.24±0.98	133.88±3.90 ✱#	89.69±3.72 ✱#♣

✱ vs. the Normal+Saline group, $p < 0.05$
# vs. the Normal+HUMSCs group, $p < 0.05$
♣ vs. the Stroke+Saline group, $p < 0.05$

Supplement Table S6. NeuN^{+} number by anti-NeuN staining brain section

Side \ Group		Normal+Saline (n=4)	Normal+HUMSCs (n=4)	Stroke+Saline (n=9)	Stroke+HUMSCs (n=10)
Cortex	Contralateral	241.25±17.57	245.25±25.39	258.00±13.03	228.20±4.66
	Ipsilateral	246.00±22.68	233.50±19.21	150.44±12.11✱#♠	232.90±5.69♣
Striatum	Contralateral	204.75±17.90	205.50±20.47	206.22±13.89	195.30±7.20
	Ipsilateral	196.00±7.04	206.75±19.81	128.44±6.61 ✱#♠	196.00±9.02♣

✱ vs. ipsilateral cortex of the Normal+Saline group, $p < 0.05$

# vs. ipsilateral cortex of the Normal+HUMSCs group, $p < 0.05$

♣ vs. ipsilateral cortex of the Stroke+Saline group, $p < 0.05$

♠ vs. contra-lateral vs. ipsi-lateral region in the Stroke+Saline group, $p < 0.05$

Supplement Table S7. Vessel length and density by Dextran-FITC staining

Side \ Group		Normal+Saline (n=4)	Normal+HUMSCs (n=4)	Stroke+Saline (n=4)	Stroke+HUMSCs (n=4)
Vessel length	Contralateral	40.66±3.44	43.56±1.73	35.09±1.78	35.71±1.73
	Ipsilateral	42.10±1.42	41.20±0.98	21.25±2.79 ✱#♠	32.50±2.03 ♣
Vessel density	Contralateral	0.24±0.01	0.25±0.01	0.23±0.01	0.22±0.01
	Ipsilateral	0.25±0.02	0.25±0.02	0.12±0.02 ✱#♠	0.24±0.02 ♣

✱ vs. ipsilateral cortex of the Normal+Saline group, $p < 0.05$
# vs. ipsilateral cortex of the Normal+HUMSCs group, $p < 0.05$
♣ vs. ipsilateral cortex of the Stroke+Saline group, $p < 0.05$
♠ vs. contra-lateral vs. ipsi-lateral region in the Stroke+Saline group, $p < 0.05$
